# Diagnostic approach, vaccination and treatment priorities for Lumpy Skin Disease in cattle: lessons from Balkan epidemics to Western Europe re-emergence (2025)

**DOI:** 10.1007/s11259-026-11190-8

**Published:** 2026-04-05

**Authors:** Eda Baldan Toker, Mevlüt Yaşar, Annamaria Pratelli, Kadir Yeşilbağ

**Affiliations:** 1 Department of Virology, Faculty of Veterinary Medicine, Bursa Uludag University, Bursa, Türkiye; 2 Department of Veterinary Medicine, University of Bari, Valenzano, Italy

**Keywords:** Lumpy Skin Disease, Treatment, Vaccination, History, Europe

## Abstract

Lumpy skin disease (LSD) remains a high-impact, transboundary infection of cattle driven by capripoxvirus transmission via mechanical vectors, causing morbidity, production losses, and trade disruption. Following the 2015–2017 Balkan epidemics, coordinated high-coverage vaccination campaigns and movement restrictions substantially reduced disease transmission across southeastern Europe. However, the recent re-emergence of LSD in Western Europe in 2025, including outbreaks reported in Italy, France, and Spain, demonstrates that regional elimination remains fragile and requires sustained preparation. This review evaluates the evidence across three practical pillars of outbreak management: diagnostic approach, vaccination strategies, and clinical/therapeutic care. Particular emphasis is placed on vaccination strategies used in field control programs, including mass vaccination, targeted and emergency (ring) vaccination, as well as the comparative performance of homologous live-attenuated Neethling vaccines and heterologous sheep and goatpox vaccines. Emerging inactivated and DIVA-compatible vaccine approaches are also discussed, together with field-level determinants of vaccine effectiveness, such as vaccination coverage, campaign timing, booster policies, cold-chain integrity, and the monitoring of adverse reactions. Based on lessons learned from European epidemics, we propose a phase-based decision-making framework that integrates rapid diagnosis, emergency vaccination, surveillance, and vector management to reduce morbidity and limit onward transmission. Because no licensed LSDV-specific antiviral therapy is currently available, treatment remains primarily supportive. It includes early detection and isolation, anti-inflammatory and antipyretic therapy, hydration and nutritional support, wound and mastitis management, and judicious antimicrobial use for secondary bacterial infections. In addition, we review emerging experimental evidence suggesting that several herbal, conventional, and antiparasitic compounds may represent promising candidates for future therapeutic approaches against LSD. Moreover, the further spread of LSD in Russia and the Middle East has been suggested to be associated with disruptions in vaccine attenuation processes, together with operational constraints affecting compliance and policy implementation in 2025.

## Introduction

Lumpy Skin Disease (LSD) is caused by Lumpy Skin Disease Virus (LSDV), a double-stranded DNA virus belonging to *the Capripoxvirus* genus within the *Chordopoxvirinae* subfamily in the *Poxviridae* family (International Committee on Taxonomy of Viruses, ICTV, 2025). In 2019, LSDV was renamed as *lumpskinpox* virus within the genus *Capripoxvirus* by the ICTV. Although LSDV shares a high degree of genomic and antigenic similarity with goatpox virus (GTPV) and sheeppox virus (SPPV), which belong to the same genus, phylogenetic analysis reveals a clear distinction between them. The disease primarily affects cattle (*Bos taurus* and *Bos indicus*), though infection can also occur in water buffaloes (*Bubalus bubalis*). This marked host specificity clarifies the target population for control strategies. At the same time, vector-mediated transmission and the virus’s environmental stability can complicate the prediction of field spread (WOAH 2022a).

The clinical spectrum of LSD ranges from fever (40–41.5 °C), lymphadenopathy (enlargement of the subscapular and precrural lymph nodes), and characteristic skin nodules (1–5 cm in size) to mastitis in cows and infertility in bulls, edema, lameness, emaciation, and secondary infections (Kumar 2011; Tuppurainen and Oura 2012; Abutarbush et al. 2015). During outbreaks, morbidity and mortality rates vary and are influenced by herd management practices, vector pressure, host immune status, and the effectiveness of control measures. Early outbreak reports from Europe (including Greece in 2015) documented a significant clinical burden, although mortality was generally lower than morbidity (Tasioudi et al. 2016). Overall morbidity commonly ranges between 5% and 45%, but may occasionally approach 100%, whereas mortality is typically below 10%, although markedly higher rates (up to ~ 40%) have been described in severe outbreaks (Coetzer and Tuppurainen 2004). Country-specific reports illustrate this variability: in Greece, outbreaks were associated with morbidity and mortality rates of 8.7% and 0.4%, respectively (Tasioudi et al. 2016), while corresponding figures in Türkiye were 12.3% and 6.4% (Şevik and Doğan 2017). However, low mortality does not diminish the impact of LSD; the disease causes significant economic losses through reduced milk production, loss of skin quality, reproductive impairment, increased labor and treatment costs, and imposition of trade restrictions. Vaccination and eradication measures account for a significant share of the total cost of outbreaks in the Balkans (vaccination, culling/stamping-out, production losses). From a clinical perspective, LSD management currently relies on standardized supportive care and the prevention of secondary complications, given the lack of a licensed antiviral therapy against LSDV.

LSDV transmission is primarily driven by mechanical transfer via hematophagous insects, while other routes appear to contribute to varying degrees, depending on ecological and husbandry conditions. The *Stomoxys calcitrans*, the best-studied vector, is consistently implicated as an efficient mechanical carrier (Chihota et al. 2003; Sohier et al. 2019; Issimov et al. 2020, 2021; Sanz-Bernardo et al. 2021; Paslaru et al. 2022). Experimental studies demonstrate that LSDV can persist for several days within the fly, particularly in mouthparts and thorax, and may be shed via regurgitated blood and feces, supporting short-term mechanical spread without evidence of viral replication within the insect (Chihota et al. 2003; Issimov et al. 2020, 2021; Paslaru et al. 2021; Sanz-Bernardo et al. 2022). Transmission modeling has further suggested a high epidemic potential for *S. calcitrans*–mediated spread, with estimated basic reproduction number (R₀) ranging from approximately 15.5 to 19.1 (Gubbins 2019; Sanz-Bernardo et al. 2021). In addition, wind-assisted introduction of infected vectors has been hypothesized as a possible mechanism for the incursion of LSD in Israel in 1989 (Yeruham et al. 1995). Several mosquito species, including *Aedes aegypti*,* Culex quinquefasciatus*, and *Anopheles stephensi*, can retain LSDV for 4–10 days, but *Aedes aegypti* is the only species for which experimental transmission to susceptible cattle has been clearly demonstrated (Chihota et al. 2001, 2003; Sanz-Bernardo et al. 2021; Paslaru et al. 2022). Transmission by *Culicoides* spp. remains unproven under experimental conditions (Chihota et al. 2003; Sanz-Bernardo et al. 2021; Paslaru et al. 2022), whereas tabanids (e.g., *Tabanus*, *Haematopota*) are capable of mechanical transmission, and field detection supports their involvement (Sohier et al. 2019; Orynbayev et al. 2021). Non-biting flies may also contribute to secondary farm-level spread (Sprygin et al. 2019; Wang et al. 2022). In addition, hard ticks are increasingly recognized as potential vectors/reservoirs: infectious viruses have been isolated from *Dermacentor marginatus* and *Hyalomma asiaticum* in Kazakhstan, and viral DNA has been detected in *Rhipicephalus/Amblyomma spp*. (Tuppurainen et al. 2015; Orynbayev et al. 2021). Experimental studies demonstrate intra- and transstadial, as well as transovarial, transmission of LSDV with overwinter survival, suggesting a reservoir role (Lubinga et al. 2014a, b, 2015; Rouby et al. 2017). Direct contact transmission is generally inefficient (Carn and Kitching 1995), although vector-free contact spread has been demonstrated experimentally using a virulent recombinant strain (Aleksandr et al. 2020). Indirect transmission may occur via contaminated fomites, water, or feed, and iatrogenic practices; the environmental persistence of the virus (lasting weeks in scabs and months in dark barns) further strengthens this risk (Tuppurainen et al. 2017; Shumilova et al. 2022; WOAH 2022b; Akther et al. 2023). Recent reviews have further emphasized that LSD epidemiology is strongly influenced by vector-driven transmission dynamics together with seasonal and environmental factors that shape arthropod abundance and movement patterns, thereby affecting local outbreak amplification and cross-border spread (Sharma and Kumar 2025; Yeşilbağ et al. 2026). Reproductive transmission is also plausible, given prolonged viral shedding in semen, and vertical transmission has been reported via the placenta, udder lesions, or milk (Irons et al. 2005; Osuagwuh et al. 2007; Annandale et al. 2014, 2019; Rouby and Aboulsoud 2016; Sudhakar et al. 2020).

The importance of LSD for Europe extends beyond its rapid epidemic spread across the Balkans. Following the Balkan-centered expansion between 2015 and 2018 (Tasioudi et al. 2016; Mercier et al. 2018; Calistri et al. 2019), the re-emergence of LSD in Western Europe in 2025 has once again demonstrated that the disease has a dynamic that is “*controllable but difficult to keep under control sustainably*”. This re-emergence underscores the need for an integrated assessment of multiple interacting components, such as (i) the seasonality and climate-related expansion of vector activity, (ii) animal movements and biosecurity gaps, (iii) field-level vaccination capacity and acceptability, and (iv) the economic/social consequences of control measures. Consistent with this perspective, the European Commission’s definition of the disease highlights LSD’s transmission, particularly through biting insects, and the substantial economic consequences of the disease, including loss of milk production, weight loss, skin damage, and reproductive disorders.

This review synthesizes current evidence on diagnostic strategies, vaccination options, and clinical management priorities for LSD in cattle, framing practical decision-making for outbreak response. Drawing lessons from the 2015–2018 Balkan epidemics and the re-emergence in Western Europe in 2025, field-determined control success factors are highlighted and integrated, and a phase-based approach to reduce morbidity and limit onward transmission is proposed. In this context, vaccination and treatment priorities were defined using a practice-oriented framework based on outbreak epidemiology, transmission risk, feasibility of field implementation, and the level of available evidence. Vaccination strategies were prioritized according to epidemiological setting (endemic, high-risk disease-free, or newly affected regions), the required speed of protection, expected vaccination coverage, and operational considerations such as vaccine availability, cold-chain integrity, and post-vaccination monitoring capacity. Treatment priorities were ranked according to immediate clinical necessity, the prevention of secondary complications, animal welfare, and the strength of current evidence, with supportive care emphasized over experimental or repurposed antiviral approaches that still lack sufficient in vivo and field validation.

## History of LSD: Africa

The disease was first reported in Zambia (formerly Northern Rhodesia) in 1929 and initially thought to result from a hypersensitivity reaction to poisoning or fly bites. However, subsequent outbreaks in Botswana (then Bechuanaland), Zimbabwe (then Southern Rhodesia), and South Africa between 1943 and 1945 confirmed its contagious nature. A widespread panzootic occurred in South Africa, lasting until 1949, affecting approximately eight million cattle and resulting in severe economic losses (Thomas and Mare 1945; Von Backstrom 1945; Diesel 1952). The disease remained confined to South Africa until 1956, after which it spread to central and eastern Africa and was subsequently reported in several African countries, including Madagascar (Buck et al. 1956). LSD was first identified in Kenya in 1957, followed by reports from Sudan in 1972 and West Africa in 1974 (Davies 1982). Therefore, the disease remained restricted to sub-Saharan Africa until 1988.

LSD was clinically recognized in Suez, Egypt, in May 1988, likely introduced through cattle imported from sub-Saharan countries. Over a period of five to six months during the summer of 1989, the disease spread to 22 of 26 Egyptian governorates, prompting the vaccination of approximately 2 million cattle with a sheeppox vaccine. The overall morbidity rate among the national cattle population was relatively low (approximately 2%), with 1,449 recorded deaths (Davies 1991). The first LSD outbreak outside the African continent occurred in southern Israel in 1989. All infected animals were culled, and vaccination of susceptible cattle with sheeppox virus successfully prevented further clinical cases (Yeruham et al. 1995). However, nearly fifteen years later, LSD re-emerged in both countries in 2006 (Brenner et al. 2006; El-Kholy et al. 2008). Until the late 1980s, LSD had mainly been confined to Africa, persisting as an endemic cattle disease for approximately six decades, following its initial discovery. Currently, the likelihood of eradicating LSD from Sub-Saharan Africa remains low, and new epidemics in previously endemic regions are still possible (Hussien et al. 2022). Recognizing its significance, the World Organization for Animal Health (WOAH) has listed LSD as a notifiable disease (List A) since 1990. It emphasizes the need for effective surveillance, control, and eradication strategies (FAO 1991).

## Recent spread of LSD: Asia

Although unconfirmed reports of LSD emerged in Kuwait and Oman between 1983 and 1986 (Alkhamis and VanderWaal 2016), the first confirmed spread of LSDV beyond Africa occurred with the 1989 outbreak in Israel as described above. In the following years, the disease became established across the Middle East through multiple incursions. Between the late 1980s and 2013, LSD was reported for the first time in several countries, including Kuwait (1991), Lebanon (1993), the United Arab Emirates (1995), Yemen (2000), Bahrain (1993 and 2002–2003), and later in Saudi Arabia and Iraq (2013) (House et al. 1990; Shimshony and Economides 2006).

Since 2011, LSD has demonstrated the potential to spread into new regions beyond Africa and the Middle East. Major outbreaks occurred across the Middle East between 2012 and 2015, with the disease reported in Türkiye, Iran, Iraq, Jordan, Azerbaijan, and even Cyprus (EFSA AHAW 2016). During the same period, the disease also spread into the Caucasus region and southern Russia (EFSA 2017).

In recent years, the disease has spread rapidly across Asia, with a major epidemic beginning in South Asia in 2019. The first significant outbreak occurred in Bangladesh in July 2019 (Hasib et al. 2021; Parvin et al. 2022), followed by reports from China (Lu et al. 2021) and India (Sudhakar et al. 2020). Throughout 2020, successive LSD outbreaks were documented in several Asian countries, including Nepal (Koirala et al. 2022), Bhutan, Sri Lanka, Vietnam (Dao et al. 2022), and Malaysia. During 2021–2022, the virus continued to spread eastward, affecting Thailand, Laos, Pakistan, and other parts of Southeast Asia, and even reaching Indonesia and Singapore (Azeem et al. 2022). More recently, LSD was reported in Korea in 2023 and in Japan in November 2024. Notably, although no cases have yet been reported in the Americas, the potential for introduction remains significant, as does the threat to currently disease-free regions such as Australia and New Zealand.

## Epidemiology of Europe

**Fig. 1:**
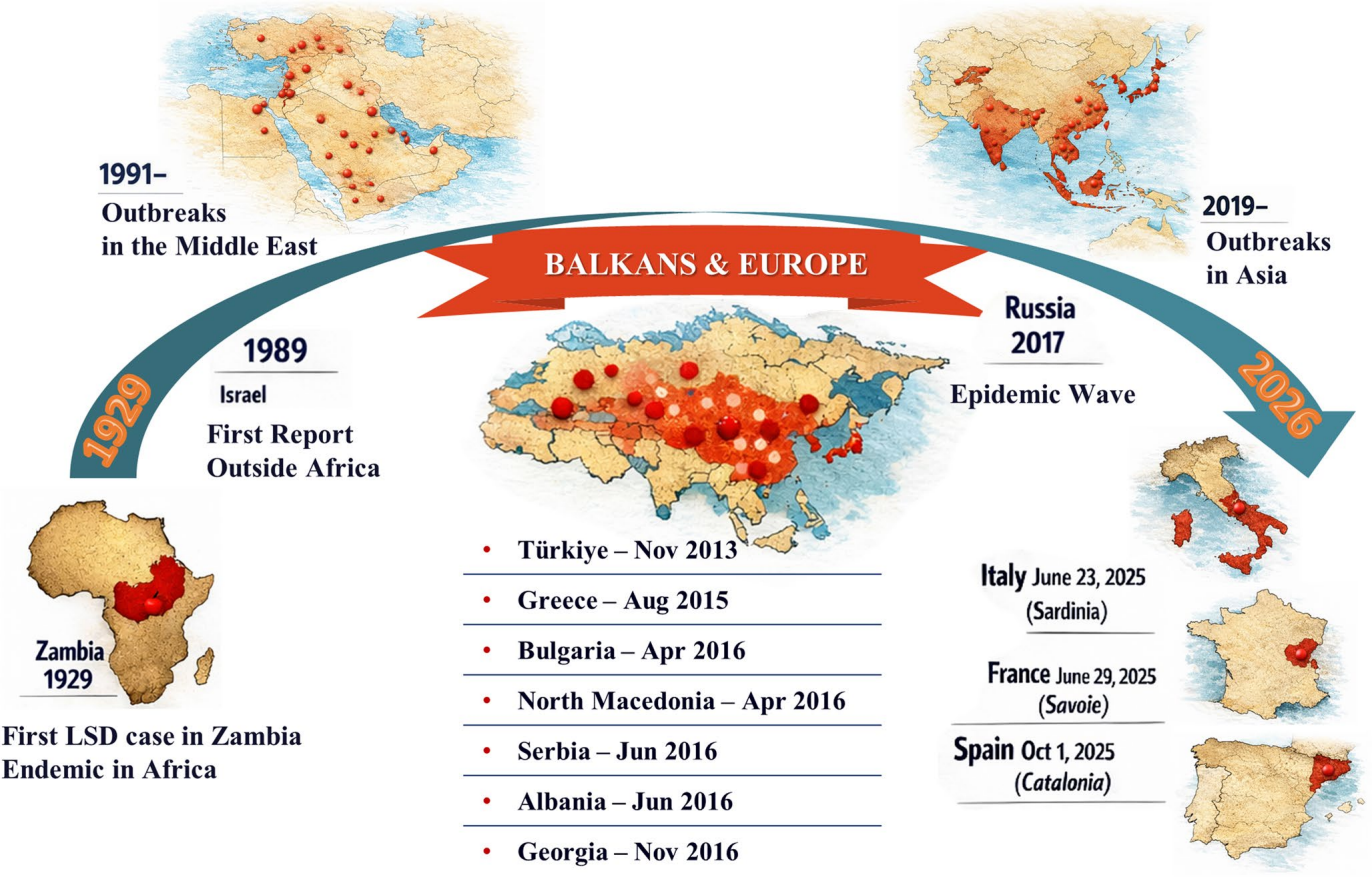
Global historical spread of Lumpy Skin Disease (LSD). The figure summarizes the geographic expansion of LSD from its first description in Zambia in 1929 through its gradual spread across sub-Saharan Africa, the Middle East, Europe, and Asia. Major epidemiological milestones are highlighted, including the first confirmed outbreaks outside Africa, the Balkan epidemics between 2015 and 2018, and the recent re-emergence of LSD in Western Europe in 2025. The timeline illustrates the progressive transboundary nature of LSD and its increasing importance for global cattle health

The LSD epidemic in Europe gained momentum with the detection of foci in Western Türkiye in May 2015, following initial entry and domestic spread in Türkiye in November 2013. The outbreak became a regional veterinary concern with its confirmation and reporting to the OIE in Greece in August 2015. Epidemiological reconstructions suggest that the main Balkan incursion most likely originated in Türkiye, reflecting its role as a geographic bridge into Southeastern Europe (Mercier et al. 2018). The initial LSD spread across the Balkans involved multiple countries, including Bulgaria, North Macedonia, Serbia, Kosovo, Albania, and Montenegro, and coincided with eastward activity, such as a substantial epidemic in the Russian Federation in 2017 and the re-emergent outbreaks in Georgia in 2018 following initial detection in 2016 (Mercier et al. 2018; Bouchemla et al. 2018; Calistri et al. 2019) (Fig. 1). Between May 2015 and August 2016, 1,092 LSD foci were recorded in Western Türkiye and the Balkans with a median spread rate of 7.3 km/week and a marked slowdown during the winter months. These data support the idea that transmission occurred through a combination of vector-borne local spread and long-distance jumps associated with animal trade/transportation (Mercier et al. 2018). Evidence from control efforts demonstrates that mass vaccination programs achieving high regional coverage can dramatically reduce the burden of outbreaks. According to the European Food Safety Authority (EFSA) reports, the number of foci in the Balkan region decreased from 7,483 in 2016 to 385 in 2017 (a reduction of approximately 95%), and no foci were reported in 2018, reflecting the continuation of coordinated vaccination programs (Calistri et al. 2019; Klement et al. 2020). The European Commission also states that this phase was successfully managed towards control and eradication through mass vaccination in the affected countries (European Commission). The “key lessons” drawn from the Balkan experience are: (i) rapid notification and surveillance, (ii) achieving high vaccination coverage through regional coordination, (iii) restriction of animal movements, and (iv) implementation of a multi-component response package during seasons of high vector activity (Tuppurainen et al. 2020).

The re-emergence of LSD in Western Europe in 2025 highlighted that the “low risk” perception following the Balkan epidemics may not be permanent. In Italy, the first confirmed outbreak was reported in Sardinia in June 2025, followed by multiple additional cases, particularly clustered on the island (WOAH-Report Information 2025a). In France, the first confirmed LSD case was reported in the Savoie region at the end of June 2025 (WOAH-Report Information 2025b). Spain reported its first LSD focus in Catalonia (near the French border, Girona) in early October 2025 (WOAH-Report Information 2025c). According to the European Commission, control measures such as culling, the designation of restricted areas, and the establishment of protection and surveillance zones, in accordance with EU legislation, were implemented at the outbreak sites. In addition, emergency protective vaccination campaigns were initiated in Catalonia and Aragon, with vaccine supplies provided from the EU’s LSD vaccine bank (European Commission). This development reinforced the “cross-border” nature of LSD risk along the France-Spain frontier during the summer and autumn of 2025. It highlighted the critical importance of data sharing, zone compliance, monitoring animal movements, and coordinating vector-control strategies.

## Diagnosis and early warning

### Clinical suspicion and differential diagnosis

Early warning for LSD typically begins with herd-level recognition of syndromic signs, most commonly fever and characteristic cutaneous nodules, sometimes accompanied by lymph node enlargement and mucosal discharges. However, clinical suspicion should be promptly followed by laboratory confirmation, as field experience shows that case finding based solely on clinical suspicion is routinely coupled with reference-laboratory testing during outbreaks (Agianniotaki et al. 2017; Amin et al. 2021). Surveillance based only on overt lesions may miss mild or subclinical infections, and targeted testing strategies have therefore been explored to improve detection sensitivity under field conditions (Aerts et al. 2021). Because several infectious and non-infectious conditions can produce superficially similar skin lesions, differential diagnosis is crucial for early detection. Reviews emphasize pseudo-lumpy skin disease (PLSD) caused by *bovine herpesvirus-2* (BoHV-2) as a frequent clinical confounder, alongside other “look-alike” dermatological conditions, such as parapoxvirus infections (e.g., pseudo-cowpox), which cannot be reliably distinguished from LSD by gross examination alone (Lanave et al. 2020; Akther et al. 2023). Case-based reports further illustrate this challenge, documenting BoHV-2 detection or isolations in cattle presenting with pseudo-LSD–type lesions, reinforcing the need for confirmatory virological or molecular assays whenever LSD is suspected (Woods et al. 1996; Brenner et al. 2009).

### Laboratory-based confirmatory diagnosis

**Fig. 2:**
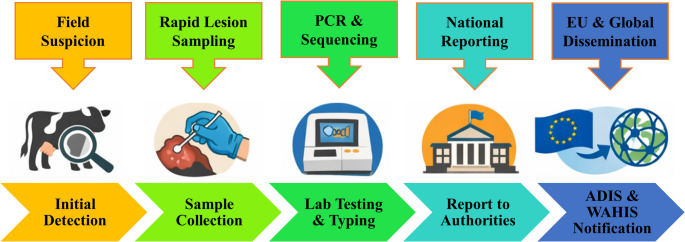
Diagnostic workflow for Lumpy Skin Disease virus (LSDV) detection and early warning. The figure summarizes the recommended diagnostic pathway following clinical suspicion of LSD. Sampling prioritizes skin nodules or scabs, which typically contain the highest viral load, followed by blood and swab samples, depending on the stage of infection. Laboratory confirmation is primarily performed using real-time PCR, while virus isolation and sequencing may be used for further characterization. Serological tests such as ELISA or virus-neutralization tests are primarily used for sero-surveillance and post-vaccination monitoring rather than for early diagnosis. Integration of laboratory confirmation with international notification systems (WOAH–WAHIS and EU ADIS) supports rapid disease reporting and cross-border epidemiological surveillance

Because LSDV loads are typically highest in cutaneous lesions, laboratory confirmation should prioritize lesion-focused sampling, complemented by blood and swab specimens depending on disease stage and operational constraints. Comparative field studies using real-time PCR have consistently shown superior detection from skin nodules or scabs compared with whole blood and some swab matrices, establishing skin as the preferred specimen for early confirmation and maximizing diagnostic sensitivity (Zeedan et al. 2019, 2024; Li et al. 2022). In practice, recommended specimen sets commonly include full-thickness skin biopsies or scabs, EDTA blood (or buffy coat when available), nasal/oral swabs, and paired sera for retrospective inference or monitoring vaccination responses; semen may be collected in specific risk contexts (Amin et al. 2021; Farag et al. 2025). The diagnostic workflow for LSDV, including recommended tests, sample types, intended use, practical considerations, and supporting evidence, is summarized in Fig. 2; Table 1.

**Table 1:** Diagnostic workflow for lumpy skin disease virus (LSDV) in cattle: recommended tests, specimen types, intended use, practical considerations, and supporting evidence

Test / method	Recommended specimen(s)	Primary purpose	When to use	Key advantages / limitations	Reference
LAMP	Lesion material (often); extracted DNA	Rapid/field-adaptable molecular confirmation	Pen-side / decentralized screening, rapid triage before sending to reference lab	Simple equipment; fast; accuracy can approach PCR; still needs local validation/QC	(Mwanandota et al. 2018)
RPA	Lesion material / extracted DNA	Ultra-rapid molecular detection	Very rapid screening in field/mobile units; early outbreak triage	Minutes-scale; low temp requirement; assay design/QC critical; often confirmed by qPCR	(Shalaby et al. 2016)
RPA + CRISPR-Cas12a (fluorescence)	Extracted DNA from clinical samples	High-sensitivity rapid detection	High-stakes rapid confirmation when qPCR access is limited and low-copy detection matters	Very sensitive/specific; reagent/workflow maturity needed; implementation-dependent	(Jiang et al. 2022)
Conventional PCR	Skin biopsies/nodules; swabs; sometimes blood	Confirmatory detection where qPCR not available	Resource-limited labs, confirmatory testing for suspected cases	Lower infrastructure than qPCR; slower/less quantitative; contamination control needed	(Zeedan et al. 2019; Pervin et al. 2023)
Multiplex / nested PCR panels	Lesion material and/or swabs	Parallel detection/typing (multiple targets)	Differential workup or when multiple agents/targets must be assessed in parallel	Efficient multi-target screening; higher complexity; strict QC required	(Bajpai et al. 2025)
Real-time PCR (qPCR/rt-qPCR)	Skin scabs/nodules (preferred); oral/nasal swabs; whole blood	Rapid confirmatory diagnosis	Index case confirmation, outbreak amplification phase (high throughput), movement-control decisions	High sensitivity/specificity & throughput; specimen choice matters (skin often highest yield); needs equipment/trained staff	(Zeedan et al. 2019; Li et al. 2022)
Sequencing / genomic characterization	Isolate or high-quality clinical DNA	Strain/variant characterization	Atypical epidemiology, suspected vaccine-like/recombinant events, cross-border investigations	Highest resolution; higher cost/infrastructure; turnaround may be longer	(van Schalkwyk et al. 2020; Krotova et al. 2022)
Virus isolation (cell culture)	Skin nodules/biopsies; sometimes swabs/buffy coat	Definitive confirmation; obtain live virus for characterization	First incursion investigations, atypical outbreaks, research/strain characterization	High value for downstream work; slow, labour-intensive; biosafety and expertise required	(Pervin et al. 2023)
Embryonated egg (CAM) isolation	Skin biopsies/nodules	Practical live-virus recovery route	When cell culture capacity is limited but virus recovery is needed	Useful alternative; time-consuming; requires egg supply/skills	(Zeedan et al. 2019)
Serology: VNT	Serum (paired sera)	Neutralizing antibody measurement	Post-vaccination monitoring, serosurveillance, retrospective exposure assessment	Reference for neutralizing antibodies; slower, labour-intensive; biosafety considerations, cross reactions	(Krešic et al. 2020)
Serology: ELISA (validated formats)	Serum	High-throughput serosurveillance	Large-scale surveillance, vaccination campaign monitoring at population level	Scalable/fast; performance depends on assay validation and context; cross reactions	(Zeedan et al. 2019; Krešic et al. 2020)
Serology: IFAT (comparative use)	Serum	Complementary antibody testing	Confirmatory/adjunct serology where ELISA/VNT results need triangulation	Useful comparator; interpretation and throughput vary	(Milovanović et al. 2019; Zeedan et al. 2019)

*LAMP: Loop-Mediated Isothermal Amplification; RPA: Recombinase Polymerase Amplification; PCR: Polymerase Chain Reaction; CAM: Chorioallantoic Membrane; QC: Quality Control; VNT: Virus Neutralization Test

#### Virological methods (reference/confirmatory)

Virus isolation remains a valuable approach for definitive confirmation and downstream characterization of LSDV. However, it is inherently slower than molecular testing and requires higher biosafety standards and laboratory capacity. Virus isolation has been successfully achieved using embryonated chicken eggs via chorioallantoic membrane (CAM) inoculation, followed by adaptation in cell culture systems (e.g., Vero cells). This approach provides a practical route for obtaining viable virus when culture facilities are limited (El-Ansary et al. 2022; Pervin et al. 2023).

#### Molecular detection (routine frontline)

For outbreak response, real-time PCR (qPCR/rt-PCR) is widely adopted as the frontline confirmatory tool due to its speed, high analytical performance, and suitability for high-throughput testing. This approach allows paired testing of lesion and blood samples from naturally infected cattle, ensuring sensitive and timely detection (Zeedan et al. 2019; Amin et al. 2021). Where multiplexing is required (e.g., to screen multiple specimen types or parallel targets), PCR panels can be integrated into the diagnostic workflow while maintaining rapid turnaround times (Farag et al. 2025).

#### Field-deployable and isothermal platforms (early warning acceleration)

To shorten the interval between clinical suspicion and laboratory confirmation, especially in decentralized resource-limited settings, loop-mediated isothermal amplification (LAMP) has been validated as a more straightforward alternative method with diagnostic accuracy reported as sensitive as or comparable to PCR in field-evaluable formats (Das et al. 2012; Mwanandota et al. 2018).

#### Serology (surveillance and post-vaccination monitoring)

All virus species within the genus *Capripoxvirus* share major neutralizing epitopes, which prevent differentiation of *Capripoxvirus* strains from cattle, sheep, or goats using conventional serological techniques. Consequently, serology is primarily used for sero-surveillance and for assessing immune responses after vaccination, rather than for early case confirmation. Despite lower sensitivity, the virus neutralization test (VNT) has been used to monitor protective antibody responses. At the same time, validated ELISA formats enable larger-scale screening (Krešic et al. 2020; Sthitmatee et al. 2023). Western blot testing using capripoxvirus-infected cell lysates can detect antibodies against viral structural proteins in the tested sera with high sensitivity and specificity, though the method is expensive and technically challenging (WOAH Terrestrial Manual 2024; Wang et al. 2026). The indirect fluorescent antibody test (IFAT) is also widely used to detect antibodies against *capripoxviruses*, supporting sero-surveillance, retrospective investigations, and post-vaccination monitoring (Zeedan et al. 2019; Krešic et al. 2020). Compared with IFAT, ELISA is better suited for large-scale sero-surveillance due to its standardization, high throughput, and reproducibility (Zeedan et al. 2019).

### EU/International notification and data flow

A robust diagnosis-and-early-warning framework explicitly links laboratory confirmation to formal notification, recognizing that reporting systems are not passive repositories, but active mechanisms for triggering regional risk management. The WOAH’s World Animal Health Information System (WAHIS) serves a dual function: (*i*) as an early warning system for immediate alert management of WOAH-listed and emerging diseases, and (*ii*) as a monitoring mechanism for periodic updates. WOAH guidance defines immediate notifications and follow-up reports as the operational foundation of the early warning function. Within the EU, notification and situational awareness are supported by the Animal Disease Information System (ADIS), which provides standardized conditions for Union-level notification and reporting under Regulation (EU) 2020/2002. ADIS is designed to document the evolution of major infectious animal diseases. Notably, the European Commission notes that ADIS and WAHIS are linked for Early Warning notifications, ensuring rapid and consistent reporting of immediate notifications and follow-up reports across borders. From an operational standpoint, an effective early-warning pipeline enables neighboring jurisdictions to adapt surveillance intensity, animal movement restrictions, and vaccination preparedness in near real-time (Fig. 2). Evidence from the 2025 EU detections demonstrates how official confirmation was rapidly translated into public risk communication and updates disease-status information through these interconnected systems (European Commission; GOV.UK, 2025).

## Vaccination strategies

Several studies have demonstrated that large-scale vaccination campaigns represent the most effective strategy for preventing the spread of LSD. Achieving herd immunity through high vaccination coverage is critical for slowing down and eventually stopping the transmission of the virus, particularly in endemic or newly affected regions. For example, a modeling study by Almuallem and Chauhan (2025) showed that increasing vaccination rates can eliminate LSD outbreaks. Conversely, quarantine measures and movement restrictions alone, although effective in reducing infection rates, cannot completely eradicate the disease. Consequently, the design of effective vaccination strategies and the achievement of high coverage levels are fundamental principles for controlling LSD and mitigating its associated economic losses (Tuppurainen et al. 2021). Recent review articles have also summarized advances in LSD vaccine development and field vaccination strategies, highlighting improvements in vaccine design, integration with diagnostic surveillance, and the strategic planning of vaccination in both endemic and newly affected regions (Farag et al. 2025; Sarkar et al. 2026).

Several types of vaccines against LSDV are currently available, with live attenuated vaccines being the most widely applied. They can be broadly divided into two categories. Homologous vaccines are derived directly from LSDV, most commonly the Neethling strain. In contrast, heterologous vaccines are based on sheeppox virus (SPPV) or goatpox (GTPV) virus, both members of the genus *Capripoxvirus*, and confer cross-protection against LSD (Ayelet et al. 2013; Gari et al. 2015). Homologous live vaccines generally induce strong and long-lasting immunity and have proven effective in controlling the disease in endemic regions (Tuppurainen et al. 2021). In Europe, Neethling-based homologous vaccines have achieved successful disease suppression. However, these vaccines may occasionally induce mild post-vaccination reactions, collectively referred to as “Neethling disease”. Symptoms may include local swelling at the injection site, small cutaneous nodules, and a transient reduction in milk yield, particularly in cattle receiving the vaccine for the first time (Katsoulos et al. 2018; Tuppurainen et al. 2021). Heterologous vaccines, derived from SPPV and GTPV, have also been widely used in several countries and are generally effective in reducing clinical severity and limiting spread, but they do not consistently achieve eradication (Ayelet et al. 2013; Gari et al. 2015). However, their effectiveness may vary depending on strain compatibility, and some reports have shown that inadequately attenuated sheep- or goatpox vaccines can induce LSD-like disease under field conditions (Tuppurainen et al. 2014; Sudhakar et al. 2022). Indeed, incomplete attenuation of vaccines produced from the Kenyan sheep and goat pox (KSGP), O-240/O-180 strains, was identified as a contributing factor in the LSD outbreak in India in 2019 (Tuppurainen et al. 2014; Sudhakar et al. 2020, 2022). Subsequent investigations demonstrated that the KSGP vaccinal strain was initially derived from LSDV and had not undergone sufficient attenuation during vaccine development (Tuppurainen et al. 2014). These findings highlight the importance of genetic stability and an adequate degree of attenuation as critical determinants of safety and performance when using live vaccines.

In addition to live vaccines, inactivated vaccines are also being developed for the control of LSD. Experimental studies indicate that, when formulated with appropriate adjuvants, inactivated LSD vaccines can provide levels of protection comparable to those achieved with live vaccines. For instance, two doses of a binary ethyleneimine (BEI) inactivated Neethling strain vaccine formulated with an oil adjuvant elicited higher neutralizing antibody titers in cattle than a single-dose live vaccine. Following a challenge with a virulent virus, vaccinated animals also exhibited significantly lower clinical scores than unvaccinated controls, and only minimal amounts of the LSDV genome were detected in internal organs, supporting the vaccine’s safety and efficacy (Hamdi et al. 2020). Similarly, other studies have reported that inactivated LSD vaccines prepared with different adjuvant formulations provide adequate protection against lethal virus challenge, although the choice of adjuvant significantly affected the strength of the immune response (Wolff et al. 2020). The main disadvantages of inactivated vaccines are their higher production cost and the requirement for multiple doses compared with live vaccines. Nonetheless, inactivated vaccines are increasingly considered a safe alternative, especially for use in disease-free regions at risk of LSD introduction.

Successful control of LSD lies in the selection of an appropriate vaccine and the implementation of effective vaccination strategies. Three principal approaches are commonly applied: (*i*) mass vaccination, (*ii*) targeted vaccination, and (*iii*) emergency vaccination. Mass vaccination involves the routine immunization of all susceptible animals in regions where the disease is endemic or the risk of introduction is high. The primary goal is to establish herd immunity by vaccinating the majority of the population within the target area. In many countries, mass vaccination campaigns are conducted annually or regionally to control LSD. For example, following the initial spread of LSD in Türkiye and neighboring Middle Eastern countries in 2013, authorities implemented large-scale vaccination programs covering all cattle in affected regions. When high coverage (exceeding 80%) is achieved and protective immunity is established, mass vaccination can interrupt long-distance transmission and prevent reintroductions (Calistri et al. 2019). Indeed, coordinated vaccination campaigns implemented after LSD outbreaks in the Balkans led to the disappearance of the disease within a few years.

Targeted vaccination involves the preventive immunization of defined target areas where the disease is not yet present but is considered at high risk, or of a limited area surrounding an outbreak (Babiuk 2018; Tuppurainen et al. 2020). ​​A related approach is vaccination of the affected herd and neighboring herds once the disease is detected. When combined with quarantine measures and movement restrictions, targeted vaccination aims to contain the virus’s spread within a confined area. This strategy also avoids unnecessary immunization of the entire susceptible population, allowing for efficient resource use, and is therefore suitable for emergency vaccination scenarios.

Emergency vaccination refers to a rapid vaccination campaign implemented as an immediate response when LSD is suddenly detected or newly introduced into a region. The primary goal is to rapidly break the chain of infection by vaccinating all susceptible animals in the affected area as early as possible during the outbreak. Emergency vaccination may be combined with other control measures, such as culling of infected herds and disinfection, or implemented independently, depending on national LSD control policy and the speed of disease spread. For example, in several European countries, including Albania, Bosnia and Herzegovina, Bulgaria, Croatia, Greece, Montenegro, North Macedonia, and Serbia, initial LSD detection was managed through culling of infected herds, coupled with emergency vaccination (ring vaccination) applied within a 3–10 km radius of the infection hotspot (WOAH 2023). This strategy aims to eliminate sources of infection while preventing spread to surrounding herds. The vaccine used in emergency campaigns must provide rapid protective immunity. Therefore, live attenuated vaccines are generally preferred as they typically induce protection more quickly and often after a single dose. However, emergency vaccination also requires systematic post-vaccination monitoring, field evaluation of vaccine performance, and timely administration of booster doses as needed (Babiuk 2018; Tuppurainen et al. 2020).

However, variations in vaccine efficacy have been observed among different LSDV vaccine strains. Field and experimental studies conducted in Africa and the Middle East indicate that heterologous Gorgan goatpox virus (GTPV)-based vaccines, particularly those derived from the Iranian Gorgan goatpox strain, can confer protection comparable to or even greater than that provided by homologous LSDV vaccines. For example, a study in Ethiopia reported that all cattle vaccinated with the Gorgan goatpox vaccine were fully protected against lethal LSDV challenge, whereas some animals immunized with the Neethling strain LSD vaccine or the Kenyan sheep-goatpox (KSGP O-180) vaccine developed clinical disease (Ayelet et al. 2013; Gari et al. 2015). The Gorgan strain vaccine induced a stronger immune response and complete clinical protection in vaccinated calves. In contrast, vaccinated animals in the KSGP and Neethling groups exhibited lower, but still detectable, rates of fever and nodule development. These results suggest that insufficient protection may arise from characteristics intrinsic to certain vaccine strains, including over-attenuation or genetic changes acquired during vaccine development and production (Gari et al. 2015). Indeed, inadequate vaccine-induced immunity has been associated with strain incompatibility between circulating field viruses and vaccine strains, suboptimal vaccine potency, and deficiencies in storage, handling, or administration. In Ethiopia, in particular, the combined use of an over-attenuated Neethling-based vaccine and the circulation of genetically diverse LSD strains have been implicated in observed field vaccination failure (Ayelet et al. 2013).

## New generation vaccine approaches and recommendations

Although currently available vaccines provide high levels of protection against LSD, ongoing research is focused on the development of next-generation vaccines that are safer, more effective, and easier to administer under field conditions. In particular, the development of DIVA (Differentiating Infected from Vaccinated Animals) vaccines has gained importance, as they address some of the limitations associated with live attenuated vaccines, including adverse reactions, risks of reversion, and challenges for surveillance in vaccinated populations. One promising approach is to incorporate a heterologous marker antigen into inactivated vaccine formulations. For example, Ronchi et al. (2024) demonstrated that the inclusion of keyhole limpet hemocyanin (KLH) as a marker antigen in an inactivated LSD vaccine enabled reliable serological differentiation between vaccinated and naturally infected animals (Ronchi et al. 2024). Experimental vaccination with the KLH-marked formulation induced a robust antibody response, and vaccinated cattle showed a marked reduction in both clinical signs and viremia following challenge with a virulent LSDV strain. These findings highlight the potential of DIVA-compatible inactivated vaccines to support both effective disease control and post-vaccination surveillance, particularly in regions aiming for eradication or disease-free status. In addition, the homologous live attenuated Lumpi-ProVac^Ind^ developed in India is another example of a next-generation vaccine. This strain, attenuated in Vero cells through 50 serial passages, has demonstrated high levels of protection in both experimental challenge studies and field applications. In controlled challenge studies, complete protection was achieved in all vaccinated animals. In contrast, field strains resulted in seroconversion rates exceeding 85% and only a very low incidence of mild post-vaccination reactions (Kumar et al. 2023a). At the same time, several approaches are available to support the DIVA strategy. The live attenuated vaccine strain, Lumpi-ProVac^Ind^, developed in India, incorporates features that support DIVA-compatible surveillance. The vaccine strain carries a unique 801-nucleotide deletion in the inverted terminal repeat (ITR) region of its genome, which enables clear molecular differentiation between vaccine and field strains. This genetic marker can be detected using a high-resolution melting (HRM)-gap qRT-PCR assay, providing a robust tool for monitoring vaccination programs and for identifying potential vaccine-induced transmission during outbreak investigations (Kumar et al. 2023b). Such marker-based differentiation represents a significant advance in balancing effective vaccination with epidemiological traceability. Complementary serological DIVA approaches are also emerging. Nokhwal et al. (2025) developed an ELISA targeting the ORF154 gene of LSDV, which provides high accuracy in distinguishing naturally infected from vaccinated animals (Nokhwal et al. 2025). This approach represents a practical solution for strengthening surveillance, especially in regions implementing large-scale or emergency vaccination programs where differentiation between infection and immunization is critical.

Another innovative approach to LSD vaccine development is the use of LSDV as a viral vector platform, enabling the generation of multivalent vaccines that confer protection against multiple cattle diseases through a single immunization. Recombinant LSD vaccines are engineered by inserting antigen genes from other important bovine pathogens into the LSDV genome, thereby inducing immunity against both LSD and additional infections. For example, recombinant vaccines expressing key glycoproteins from Rift Valley Fever have demonstrated protective immune responses against both LSD and Rift Valley Fever in preclinical and clinical studies (Wallace et al. 2020). Similarly, LSDV-based vectors have been explored for protection against rabies (Aspden et al. 2002) and rinderpest (Ngichabe et al. 1997), showcasing the platform’s versatility. Similarly, combination vaccines incorporating LSDV with other antigens have been developed and tested. For example, an inactivated oil-adjuvanted combination vaccine against LSDV and Bluetongue virus elicits strong neutralizing antibody titers against both diseases in vaccinated animals (Es-sadeqy et al. 2021). Such integrated vaccination strategies hold considerable promise for comprehensive control of epidemic diseases in livestock, while optimizing resource use and field vaccination logistics.

Emerging vaccine technologies and genetic engineering approaches are increasingly being explored in the quest for next-generation vaccines against LSD. These include recombinant protein subunit vaccines, viral vector vaccines such as those based on adenoviruses or capripoxviruses, and cutting-edge mRNA vaccine platforms. Although no LSD vaccine utilizing these advanced technologies has yet received full regulatory approval, promising results from their application in other veterinary and human diseases provide a strong rationale for their adaptation to LSD control. Among these, mRNA vaccines, in particular, have garnered attention for their rapid production and safety advantages; however, their ability to induce effective cellular immunity against DNA viruses such as LSD remains under investigation.

Improving the existing vaccines remains a key goal in LSD control efforts. For example, comprehensive genetic characterization of live attenuated vaccine strains is recommended to ensure their stability and to enable updating with more immunogenic variants when necessary (Gari et al. 2015). Optimizing vaccine dosages is also an important part of the investigation; a study demonstrated that increasing the dose of a heterologous live vaccine significantly enhanced the humoral immune response but was also associated with a higher incidence of adverse effects. Therefore, identifying an optimal dose-response balance that maximizes protection while minimizing adverse effects is crucial (Zhugunissov et al. 2020). Similarly, training field teams in proper vaccine storage (cold chain) and administration techniques plays a critical role in improving vaccine efficacy under field conditions (Ayelet et al. 2013). Finally, recommendations for vaccination strategies emphasize sustainability and ongoing monitoring. Long-term control of LSD is more effectively achieved through multi-year vaccination programs tailored to the local epidemiological contexts, rather than isolated campaigns. For example, in endemic areas, annual booster vaccinations (especially for breeding bulls and pregnant cows) are recommended to maintain protective immunity. Calves should ideally be vaccinated at approximately 3–4 months of age to overcome interference from maternally derived antibodies and ensure timely acquisition of active immunity (Tuppurainen et al. 2015). Modern LSD control programs should also include serological surveillance and, if necessary, molecular typing of breakthrough infections to assess vaccination performance. The integration of DIVA assays that differentiate between vaccine and field strains is strongly recommended as part of comprehensive vaccination and disease management strategies (Tuppurainen et al. 2021; Ronchi et al. 2024).

## Treatment strategies

Current treatment for LSD primarily focuses on symptomatic and supportive care. In the absence of licensed antiviral therapies, management aims to alleviate clinical signs, prevent complications, and promote recovery. Appropriate broad-spectrum antibiotics (penicillin, cephalosporin, tetracycline, or fluoroquinolone) are commonly administered for 5–7 days to counteract bacterial superinfections. Topical antiseptic sprays and ointments are applied to skin abrasions and wounds to reduce the risk of superinfection and myiasis during wound healing, and repellent sprays are recommended for severe nodular lesions. Supportive treatment also includes anti-inflammatory and analgesic medications to minimize pain, as well as antihistamines to control edema and allergic reactions (Salib and Osman 2011). Febrile or agitated animals may receive antipyretics (e.g., paracetamol), vitamin B complexes to stimulate appetite, and cardiac tonics and metabolic support as needed. In severe cases, intravenous fluids are also used to prevent dehydration and encourage feeding (Abutarbush et al. 2015). A case series from India documented full recovery within two weeks in six cattle treated weekly with antibiotics (oxytetracycline), anti-inflammatory drugs (NSAIDs), antiparasitic drugs (ivermectin), and vitamin supplements (Smriti et al. 2023).

Alternative therapeutic approaches for LSD are increasingly being investigated through phytotherapy and traditional methods. Experimental studies have demonstrated antiviral activity of several plant-derived compounds against capripoxviruses. Bhanuprakash et al. (2008) showed that leaf extracts of *Acacia arabica* and *Eugenia jambolana* inhibited goatpox virus (GTPV) replication by up to 99% (Bhanuprakash et al. 2008). More recently, Burranboina et al. (2022) reported that phytochemicals derived from *Leucas aspera* leaf extract exhibited potent inhibitory activity against capripoxvirus polymerase and major envelope protein P32, suggesting a plausible molecular basis for antiviral effects (Burranboina et al. 2022). These findings suggest the hypothesis that plant-based antivirals may hold promise against capripoxviruses, including LSD. In addition to in vitro evidence, herbal-based supplements have also been reported to alleviate clinical symptoms and accelerate recovery. A field study conducted in India assessed the efficacy of a polyherbal formulation consisting of an oral powder, topical spray, and ointment commercially known as LUMPY-NIL, in cattle affected with LSD (Kumar et al. 2025). In this study, 52 cattle received LUMPY-NIL herbal powder orally twice daily, a dermal spray was applied to closed nodules, and an ointment was used to open lesions. Treated animals reportedly showed alleviation of clinical signs and accelerated recovery, suggesting a potential role for such formulations as adjuncts to conventional supportive therapy. Results from the field evaluation showed that the formation of new nodules ceased within 2–4 days of initiating treatment, while systemic clinical signs such as fever, lethargy, and anorexia resolved within 1–3 days. Open wounds healed rapidly without evidence of secondary infection, and most cutaneous nodules had dried and fallen off by approximately day 15. Treatment lasted 7–21 days, depending on disease severity. No significant side effects were observed, and milk production fully returned to baseline levels by day 15. In addition to commercially formulated herbal products, ethnoveterinary practices are employed in specific regions for the management of LSD lesions. For example, in Zambia, traditional breeders use herbal mixtures prepared from the roots and bark of *Cassia abbreviata* and *Trema orientalis*, either administered orally to sick animals or applied externally to affected skin (Syakalima et al. 2018). However, the efficacy and safety of such treatments have not been rigorously evaluated under controlled experimental conditions, and they are therefore not routinely recommended within evidence-based veterinary practice.

Bee venom has long been used as a therapeutic agent in medicine. It is derived from the honeybee (*Apis mellifera L.*). It contains several biologically active compounds, including melittin, phospholipase A2, and apamin, which are known to exert antimicrobial, anti-inflammatory, and antiviral effects. A study investigating the Egyptian strain of LSDV demonstrated that bee venom exhibited measurable antiviral activity in embryonated chicken eggs without inducing observed toxicity (Kamal 2016).

Ivermectin, an FDA-approved antiparasitic drug, has demonstrated antiviral activity. In vitro, ivermectin inhibits the nuclear import of viral proteins by blocking the importin α/β-mediated transport pathway (Wagstaff et al. 2012). Toker et al. (2022) demonstrated in vitro that ivermectin at a concentration of 2.5µM reduces LSDV replication by approximately 3 logs, showing particular effectiveness during the viral attachment and penetration stages (Toker et al. 2022). Beyond repurposed pharmaceuticals, nanotechnology-based antiviral approaches are also being investigated. Nanoparticles, such as Silver Nanoparticles (AgNPs) and Zinc Oxide Nanoparticles (ZnONPs), are of interest due to their small size, high surface area, and capacity to interact directly with viral particles. ZnONPs have been shown to exert antiviral activity against LSDV by binding to viral envelope proteins, inducing structural damage, and suppressing viral replication in Madin–Darby bovine kidney (MDBK) cells (El-Bagoury et al. 2024).

Currently, no clinically approved antiviral treatment is available for LSD. However, experimental studies on ivermectin, bee venom, herbal extracts, and nanoparticle-based formulations have demonstrated promising antiviral potential in vitro. While these findings underscore the potential of novel and repurposed agents as adjunct or future therapeutic options, their safety, pharmacokinetics, and clinical efficacy must be rigorously evaluated through controlled in vivo studies and well-designed field trials before any recommendation for practical use in cattle can be made.

## Strategies for managing the LSD outbreaks

Control of LSD outbreaks depends on a coordinated implementation of multiple control measures, rather than reliance on any single intervention, including movement restrictions, enhanced biosecurity, disinfection of premises, zoning, culling (or stamping out) of infected herds when required, vector control, and, where appropriate, emergency and protective vaccination (Table 2). Recent modelling approaches also support the implementation of combined control strategies, demonstrating that the coordinated application of vaccination, vector control, and isolation measures can significantly reduce LSD transmission and the persistence of outbreaks (Alanazi et al. 2026). Field experience in Southeastern Europe during the 2015 and 2017 epidemics demonstrated that the combined implementation of these measures is critical for effective outbreak suppression; gaps in vaccination coverage were associated with prolonged transmission and delayed control. Movement restrictions affecting animals, vehicles, equipment, and personnel represent one of the most rapidly deployable tools and are designed to reduce contact network between infected and susceptible holdings. During the Balkan outbreaks, the enforcement of movement controls, combined with active surveillance and zoning strategies, acted synergistically with mass and emergency vaccination campaigns to limit both local spread and long-distance dissemination of the virus (EFSA 2018; Calistri et al. 2019; Tuppurainen et al. 2020). However, because LSD transmission is largely vector-mediated, a movement restrictions approach alone is unlikely to be sufficient for adequate control. Facility-level biosecurity measures, including controlled access to premises, restrictions on shared equipment, and rigorous cleaning and disinfection protocols, are essential complementary interventions, especially to reduce the risk of indirect/iatrogenic transmission (Tuppurainen et al. 2020, 2021). While stamping out is sustainable in LSD, its implementation must be carefully assessed for economic sustainability and feasibility. A quantitative evaluation of disease control costs in Balkan countries demonstrated that vaccination and eradication/culling measures accounted for a substantial share of total outbreak-related expenditures during the 2016–2017 period, with considerable variation between countries (Casal et al. 2018). These findings suggest that culling decisions should not be based solely on epidemiological effectiveness but must also account for “field realities”, including compensation schemes, logistical capacity, and farmer compliance (Casal et al. 2018; Tuppurainen et al. 2020).

**Table 2:** Feasibility matrix for Lumpy Skin Disease outbreak control measures in Europe

Bundled measure	Expected epidemiological impact	Cost & resource intensity	Social acceptability	Practical mitigation / implementation tips
Movement control + zoning/buffer areas	High (prevents long-distance jumps; focuses control geographically)	Medium–High (enforcement, tracing, mapping)	Moderate (declines with duration)	Risk-based zones; transparent criteria for lifting; shared maps; synchronized cross-border rules
Farm biosecurity + cleaning/disinfection	Medium–High (reduces indirect/iatrogenic spread; supports all measures)	Low–Medium (training + routine supplies)	Generally high if practical	Simple checklists/SOPs; audits + feedback; prioritize high-traffic farms; “pre-clean then disinfect” protocols
Integrated vector management	Medium–High in peak season (reduces short-range pressure)	Medium (repeat treatments; labor)	Variable (residue/resistance concerns)	Combine chemical + environmental; seasonal action plan; rotate where appropriate; focus on hotspots and peak months
Vaccination strategy	High–Very high if coverage is high and timely	High–Very high (vaccine supply, cold chain, teams, monitoring)	Moderate–High (trust dependent)	Pre-position stock; micro-planning by zone; mobile teams; real-time coverage monitoring; proactive adverse-event communication
Stamping-out (culling) + compensation logistics	Potentially high early-source removal, but context-dependent	Very high (culling ops, disposal, compensation)	Often low/contested	Clear humane protocols; transparent triggers; fast compensation; combine with vaccination alternatives where policy permits
Surveillance + rapid laboratory confirmation	High (speed drives all downstream decisions)	Medium (lab throughput, transport, staffing)	High if timely and accessible	Decentralize sampling; fast-track index cases; standardized reporting templates; link national–EU–WAHIS reporting
Risk communication + stakeholder governance	High indirect (compliance, early reporting, trust)	Low–Medium (staff time/outreach)	Determinant of overall success	Single message framework; two-way channels; local leaders; rapid myth-busting; “why-this-measure” explanations
Cross-border coordination	High for transboundary risk reduction	Medium	High institutionally; variable locally	Joint dashboards; synchronized vaccination windows; shared SOPs; periodic cross-border briefings

SOP: Standart operational procedure

The table outlines the principal control measures recommended during LSD outbreaks, including movement restrictions, zoning, vaccination strategies, biosecurity measures, vector control, and stamping-out policies where applicable. These interventions are typically implemented in combination and adapted to the epidemiological context of the outbreak. Evidence from the Balkan epidemics demonstrates that coordinated application of these measures, particularly high-coverage vaccination and movement control, can substantially reduce transmission and facilitate outbreak containment.

In LSD control, vector management is not a “secondary” measure, but a critical determinant of transmission intensity, especially during warm seasons when vector activity peaks. Experimental and modeling studies have shown that hematophagous insects can mechanically transmit LSDV and that epidemic dynamics are susceptible to vector–host interactions, providing strong justification for integrating vector control into comprehensive outbreak response packages (Tuppurainen et al. 2021; Sanz-Bernardo et al. 2021). In practice, vector management strategies are typically structured along two axes: (*i*) direct interventions, including insecticide/repellent applications on animals/shelters, and (*ii*) environmental management measures, such as improved manure and waste handling, waterlogging reduction, and shelter improvements. Evidence from field experience indicates that vector control is most effective when implemented alongside vaccination and movement restrictions, reinforcing a multi-layered approach to LSD containment (Tuppurainen et al. 2021).

Zoning, including the establishment of protection, surveillance, and buffer zones, enables risk-based resource allocation and the implementation of “spatially” targeted movement restrictions. During the Balkan LSD outbreaks, the European Food Safety Authority (EFSA) demonstrated that most outbreaks clustered geographically, with transmission persisting longer in areas with incomplete vaccination coverage; therefore, zone-based surveillance and vaccination planning have practical value (EFSA 2018; Calistri et al. 2019). In cross-border risk management contexts, the effectiveness of zoning is further enhanced by synchronized surveillance activity, coordinated movement control policies, and timely data sharing between neighboring regions and countries. Such coordinated approaches are essential to reduce the risk of re-introduction and to support sustainable regional control of LSD (Calistri et al. 2018; Tuppurainen et al. 2020).

## Conclusion

Although this review synthesizes current evidence on the diagnosis, vaccination, and management of LSD, several limitations should be acknowledged. First, the available literature on LSD epidemiology and control strategies is heterogeneous, encompassing experimental studies, field observations, modelling analyses, and review articles, which may differ in their methodological designs and regional contexts. Second, many studies investigating potential antiviral or alternative therapeutic approaches remain limited to in vitro investigations or small-scale experimental trials; therefore, their clinical relevance under field conditions requires further validation. Finally, the rapidly evolving epidemiological situation of LSD, particularly the recent emergence of outbreaks in previously disease-free regions, suggests that additional data on transmission dynamics, vaccine performance, and control strategies are likely to become available in the near future.

In conclusion, effective control of LSD outbreaks in Europe depends on the early deployment of an integrated bundle of interventions rather than on any single measure. This package includes movement controls and zoning, strengthened farm-level biosecurity and disinfection, targeted stamping out where mandated and operationally feasible, seasonally intensified vector management, and rapid protective (ring) vaccination. Looking beyond the 2025 re-emergence, priorities for 2026 and beyond should transition from reactive outbreak response to sustained preparedness. Key elements include: (*i*) harmonized EU-wide contingency planning with pre-positioned vaccine banks, trained surge teams, and validated cold-chain logistics; (*ii*) strengthened laboratory and field surveillance that integrates risk-based zoning with rapid confirmatory testing and, when needed, genomic investigation to inform policy; (*iii*) institutionalization of integrated vector management as a seasonal public-good intervention rather than an ad hoc farm-level measure, and *(iv*) improved feasibility through transparent compensation schemes and stakeholder-centered risk communication to protect early reporting and compliance. From a therapeutic perspective, priorities should include the development of harmonized, evidence-graded supportive care protocols and well-designed field trials to evaluate promising candidate antivirals or adjunct interventions, given the current absence of a licensed LSDV-specific treatment. Finally, medium-term research and policy should prioritize DIVA-compatible vaccination strategies, standardized metrics for field vaccine effectiveness, and cross-border data-sharing protocols that allow real-time alignment of control zones and movement measures across neighboring jurisdictions.

## Data Availability

All data generated through the review process is presented within the manuscript.
